# Impact of Alcohol Intoxication on Mortality and Emergency Department Resource Use in Suicidal Patients

**DOI:** 10.5811/westjem.48788

**Published:** 2026-01-03

**Authors:** Kevin Skoblenick, Esther Yang, Michael P Wilson, Erik Youngson, Brian H Rowe

**Affiliations:** * University of Alberta, Department of Emergency Medicine, Edmonton, Alberta, Canada; † University of Alberta, Department of Psychiatry, Edmonton, Alberta, Canada; ‡ University of Alberta, Neuroscience and Mental Health Institute, Edmonton, Alberta, Canada; § Alberta SPOR SUPPORT Unit, Data and Research Services, Edmonton, Alberta, Canada; ¶ Alberta Health Services, Provincial Research Data Services, Edmonton, Alberta, Canada; || Virginia Tech Carilion School of Medicine, Department of Emergency Medicine, Roanoke, Virginia; # Virginia Tech Carilion School of Medicine, Department of Psychiatry, Roanoke, Virginia; §§ University of Alberta, School of Public Health, Edmonton, Alberta, Canada

## Abstract

**Introduction:**

In North America, suicide ranks among the top causes of death in individuals 15–60 years of age. In this study we aimed to determine whether an emergency department (ED) presentation for suicidal behaviors accompanied by acute alcohol intoxication was associated with increased six-month suicide or all-cause mortality compared to non-intoxicated presentations of suicidal behaviors.

**Methods:**

We performed a retrospective cohort study of adults (≥ 18 years) presenting to 16 EDs in Alberta, Canada, between April 2011–March 2021. Suicidal attempt or self-harm was identified via International Classification of Diseases codes, 10th Rev, Canadian Enhancement (ICD-10-CA). Patients were classified as acutely intoxicated if they had relevant ICD-10-CA codes or a blood alcohol concentration ≥ 2 millimoles per liter (9.2 milligrams per deciliter). We excluded patients who died on arrival, were transferred, or were non-residents. The primary outcome was suicide-specific mortality at six months; secondary outcomes included all-cause mortality, use of involuntary holds, psychiatric consultations, admissions, and ED return visits. Median differences with 95% confidence intervals and unadjusted odds ratio (OR) with 95% CI were reported for continuous and categorical variables, respectively.

**Results:**

Among 58,051 suicidal or self-harm patients, 17,488 (30%) were classified as intoxicated. Six-month suicide mortality was similar between intoxicated and non-intoxicated groups (0.3% each; adjusted sub-distribution hazard ratio = 0.98 [95% CI, 0.73–1.38]), indicating no significant association between alcohol intoxication and suicide-specific death. Intoxicated patients were more often male (58% vs 52%; OR 1.26 [1.22–1.31]), arrived by ambulance (70% vs 50%; OR 2.32 [2.23–2.41]), and were more frequently placed on involuntary holds (26% vs 16%; OR 1.92 [1.83–2.00]). They had fewer hospital admissions (10.8% vs 15.4%; OR 0.63 [0.60–0.67]), longer ED stays (411 vs 277 minutes; median difference = 134 minutes [127.7–140.3]), and higher ED return rates at 30 days (19.8% vs 18.3%; OR 1.10 [1.05–1.15]) and six months (45.8% vs 42.1%; OR 1.16 [1.12–1.20]).

**Conclusion:**

Acute alcohol intoxication among ED patients presenting with suicidal behaviors was not independently associated with higher six-month suicide mortality. Patients with acute alcohol intoxication had increased use of involuntary holds, longer lengths of stay, and more frequent ED return visits. Future work should explore other psychosocial and clinical factors, including substance use and psychiatric comorbidities, that may influence outcomes beyond the acute setting.

## INTRODUCTION

Death by suicide is an international public health concern, especially among younger adults in their prime years of life. In North America, it ranks among the top causes of death in individuals 15–60 years of age, posing substantial societal and economic costs.^1^,^2^ Alcohol use disorder (AUD) is widely recognized as a risk factor for suicidal ideation, attempts, and completion.^3^–^6^ A population-based study in the United Kingdom found that alcohol-related hospital admissions conferred a markedly higher risk of suicide (hazard ratio [HR] 26.8) over six years.^6^ While a recent systematic review confirmed this association, the authors also noted a lack of research focusing on emergency department (ED) settings.^7^

Accurately identifying individuals at highest risk of suicide can be challenging, particularly when distinguishing acute risk factors, such as intoxication, from chronic comorbidities such as established AUD or depressive disorders.^8^,^9^ Although a study from Finland suggested that patients presenting to an ED with suicidal behavior and acute intoxication have lower six-month suicide mortality,^10^ other work indicates that acute alcohol use increases the likelihood of a suicide attempt, but does not clarify whether it affects subsequent deaths by suicide.^11^,^12^ Postmortem analyses have similarly yielded mixed findings on the prevalence of alcohol use among suicide decedents, further complicating the question of causation.^13^

To address the gap between well-documented chronic AUD risk and less-studied acute intoxication, we aimed to determine whether acute intoxication at the time of ED presentation for suicidal behavior increased six-month mortality (both suicide-specific and all-cause) and to describe ED transitions in care, including admissions, involuntary holds, and return visits. By leveraging a large, population-based database from an entire Canadian province of 4.3 million people, we sought to provide a more nuanced perspective on the role of acute alcohol intoxication in short-term outcomes among ED patients presenting with suicidality.

## METHODS

### Study Design and Setting

We performed a retrospective, cohort study using linked administrative databases from 16 EDs across two major catchment zones in Alberta, Canada. Each ED contributes standardized data on patient presentations to provincial databases. The University of Alberta Health Research Ethics Board approved this project (Pro00095789). The need for individual patient consent was waived due to the retrospective nature of the study.

### Selection of Participants

All adult patients (≥ 18 years of age) presenting to one of the participating EDs with suicidal attempt or self-harming behavior between April 1, 2011–March 31, 2021 were eligible. We identified cases using *International Classification of Diseases, 10^th^ Revision, Canadian Enhancement* (*ICD-10-CA*) codes for suicidal attempt or self-harm (X60-X84, Y10-Y34, and X40-X49) in the first three diagnostic fields of the National Ambulatory Care Reporting System (NACRS). These codes have been previously validated to reliably identify patients with suicidal attempt (sensitivity of 44.8% [95% CI, 40.2–49.4] and specificity of 96.6% [95% CI, 95.9–97.2]) and self-harm behavior (sensitivity of 40.0% [95% CI, 36.2–43.9] and specificity of 98.2% [95% CI, 97.7–98.7]).^14^ The low sensitivity of these codes is an inherent challenge in all studies on suicide and is discussed further in the “Limitations” section. We excluded individuals who arrived in the ED but died immediately upon arrival, those transferred from another facility for admission or specialized care, and those not covered by the Alberta Health Care Insurance Plan (AHCIP) due to an inability to link them with other databases. For patients with multiple qualifying ED visits, only the first presentation during the study period was included. We excluded any ED encounters that could not be linked across both the NACRS and Edmonton/Calgary systems or had missing data.

**Population Health Research Capsule**

What do we already know about this issue?

*Chronic alcohol use disorder increases suicide risk, but mortality of patients presenting both intoxicated and suicidal to the emergency department (ED) is unknown*.

What was the research question?

*Does acute alcohol intoxication increase 6-month suicide mortality in suicidal ED patients?*

What was the major finding of the study?

*Six-month suicide mortality was 0.3% in both intoxicated and sober groups (adjusted hazard ratio 0.98, 95% CI, 0.73*–*1.38; P = .94)*.

How does this improve population health?

*These findings clarify that acute intoxication alone does not increase short-term suicide risk, but this patient group requires more ED resources*.

Patients were classified as acutely intoxicated if they met at least one of the following criteria:

- An *ICD-10-CA* code specifically indicating acute alcohol use or intoxication (F10.0, R78.0, T51.0, X45, X65, Y15, Y90, Y91). These codes do not capture chronic AUD but rather acute effects or poisoning.
- A blood alcohol concentration (BAC) ≥ 2 millimoles per liter (mmol/L) (9.2 milligrams per deciliter (mg/dL]) recorded during the ED encounter.

Given that not all patients underwent BAC testing and that measured levels can be influenced by time-to-test, this study included both clinical coding and laboratory data to minimize misclassification. There is the possibility that patients with alcohol intoxication might have been missed if neither code nor laboratory confirmation were available; similarly, not all coded patients may have had measured BAC. The value of 2 mmol/L (9.2 mg/dL) is the lowest detectable BAC with the assay used in Alberta. As the mean serum alcohol clearance occurs at a rate of 2–7 mmol/L per hour,^15^ it is unlikely that a patient would be clinically diagnosed as intoxicated with alcohol if their BAC was < 2 mmol/L. A common protocol in Alberta EDs is to measure BAC in those patients with suicide attempts or altered levels of consciousness. This helps guide the emergency physician in reassessing and refining their differential diagnosis. Breathalyzers are rare in Canadian EDs, are exclusively used by law enforcement, and their results are not part of the medical record.

### Outcomes

The primary outcome was death by suicide within six months of the index ED visit, identified from provincial Vital Statistics records using *ICD-10-CA* underlying cause of death codes X60–X84. The six-month time frame was selected as it is the most commonly used period of time in studies regarding suicide behavior^16^ and represents the most at-risk period following an ED visit for suicidality.^17^,^18^ Secondary outcomes included all-cause mortality at 180 days, the proportion of ED patients placed under an involuntary mental health hold (Form 1Admission Certificate) within ±24 hours of their ED visit, the proportion of ED visits receiving a psychiatry consultation, hospital admission rates (encompassing both psychiatric and medical admissions), and the frequency of return ED visits at 30 days and at 180 days.

### Data Sources and Linkage

We obtained data for this study from multiple administrative sources. The NARCS provided records for all ED encounters, including up to 10 diagnostic fields including most of the cohort data. The data undergoes chart abstraction by trained health record abstractors who follow guidelines set forth by the Canadian Institute for Health Information, while other information is interfaced directly from electronic health records, all of which are routinely used for administration research purposes in Alberta and Canada. Practitioner Claims data contained physician billing information and captured the use of involuntary hold codes (SOMB 08.12A). The Emergency Department Information System (EDIS) and the Regional ED Information System (REDIS) supplied timestamps related to patient arrival, discharge, and any consultations. All patients seen in Canadian EDs receive a 5-level Canadian Triage and Acuity Scale (CTAS) score (1 = resuscitation; 2= emergent; 3 = urgent; 4 = less urgent; and 5 = non-urgent) reflecting their severity at presentation and the timing of assessment. We used Alberta’s Provincial Laboratory Data used to identify documented blood alcohol concentrations, while Vital Statistics provided information on dates and causes of death. Demographic data were obtained from the AHCIP registry (Provincial Registry). Patient information across these databases was linked deterministically via patient health card numbers. No manual chart review was performed. Any retrospective review is inherently subject to multiple sources of bias.^19^,^20^ Through the administrative design of this study’s database, the automated and electronic nature of data entry, and the expertise of those performing data extraction and analysis, we sought to mitigate potential biases in the 10 areas where they are most likely to arise, as described by Kaji et al.^21^

### Statistical Analysis

We described categorical variables with percentages, whereas continuous variables were reported with means and standard deviations for normally distributed variables or medians and interquartile ranges for non-normally distributed variables, as appropriate. For this study population, we compared the differences in those variables between patients with and without alcohol intoxication. Median differences with 95% confidence intervals and unadjusted odds ratio (OR) with 95% CI were reported for continuous and categorical variables, respectively.

We used the Fine and Gray multivariable competing risk model to estimate the hazard ratio (HR) of death from suicide within 180 days of the index ED visit.^22^ In this model, death from all-cause was considered as a competing risk since it eliminates the risk of death from suicide. We calculated the follow-up time as the number of days between the index ED visit date and the death date within 180 days or was censored at 180 days if the patient did not die. Univariate and multivariable competing risk models were run for all pre-selected variables of interest including age, sex, involuntary hold status, and presence of alcohol intoxication. We reported the unadjusted and adjusted sub-distribution HRs and the corresponding 95% CI. Other key risk factors (eg, comorbid psychiatric diagnoses, other substance use) were not reliably available in these administrative data and could not be included. We conducted all data analyses in SAS v9.4 (SAS Institute, Inc., Cary, NC). A *P*-value of < 0.05 was considered significant.

## RESULTS

**Figure 1:**
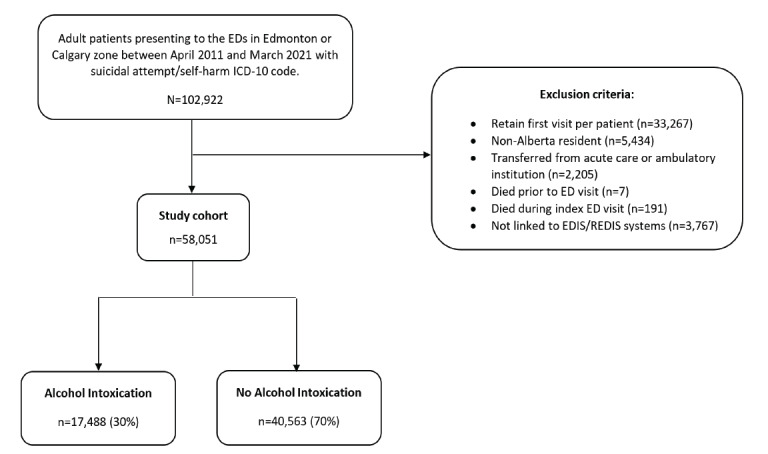
Flowchart showing derivation of the study cohort from 102,922 ED presentations for suicidal behavior to the final analytic sample of 58,051 patients, based on administrative data from 16 EDs in Alberta, Canada (2011–2021). *ED*, emergency department. *EDIS*, Emergency Department Information System; *ICD-10*, International Classification of Diseases; *REDIS*, Regional Emergency Department Information System.

During the 10-year study period, 102,922 patients ≥ 18 years of age presented to participating Alberta EDs with diagnostic codes indicating a suicidal attempt or self-harm. Of these, 44,871 were excluded for reasons such as non-Alberta residency, death upon arrival, transfer from another institution for admission, missing data, or missing linkage information, leaving 58,051 eligible patients for analysis (Figure 1). Among the final cohort, 17,488 (30%) were identified as acutely intoxicated with alcohol based on our case definition.

Baseline demographic and clinical characteristics are provided in Table 1. The overall median age was 34 years (IQR 24–48), and 53.5% of the patients were male. Compared with those who were not intoxicated, patients in the alcohol-intoxicated group more frequently arrived by ambulance (69.2% vs 49.3%; OR 2.32 [95% CI, 2.23–2.41]) and were more often male (57.5% vs 51.8%; OR 1.26 [95% CI,1.22–1.31]). There was minimal difference in CTAS distribution between the two groups, with most patients triaged as CTAS levels 2 or 3.

A total of 671 patients (1.2% of the full cohort) had an ED visit for suicidal ideation (*ICD-10-CA* R45.8) within the six months preceding their index visit for self-harm or suicide attempt. This prior visit for suicidal ideation was more common among patients who were intoxicated at the time of their index presentation compared to those who were not (1.5% [256/17,488] vs 1.0% [418/40,563]; P < .001).

**Figure 2:**
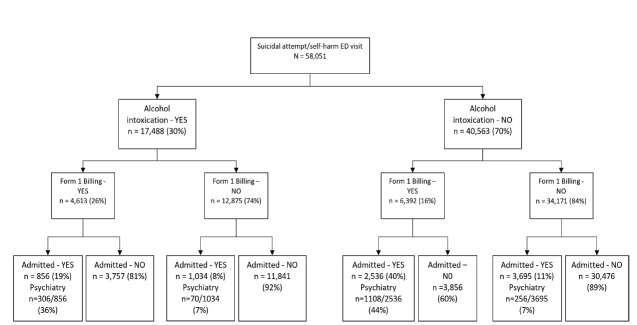
Breakdown of emergency department disposition outcomes among patients presenting with suicidal behavior, stratified by alcohol intoxication status, including rates of involuntary mental health hold, hospital admission, and psychiatric admission, in a retrospective cohort study of 58,051 patients across 16 EDs in Alberta, Canada (2011–2021). *FORM 1*, involuntary mental health hold.

The median ED length of stay (LOS) was longer for the intoxicated group (411 minutes [IQR 255–640]) than for the non-intoxicated group (277 minutes [IQR 158–486]). Management patterns in the ED differed between groups, as shown in Table 2. Among intoxicated patients, 26% were placed under an involuntary mental health hold, compared with 16% of those who were not intoxicated (OR 1.92; 95% CI, 1.83–2.00). Psychiatric consultations were recorded for 15.8% of the intoxicated group vs 12.6% of the non-intoxicated group (OR 1.31; 95% CI, 1.24–1.37). Although a higher proportion of intoxicated patients received an involuntary hold, they had a lower hospital admission rate (10.8% vs 15.4%; OR 0.63; 95% CI, 0.60–0.67). Among patients who received an involuntary hold, the admission rate was 19% for the intoxicated group vs 40% for the non-intoxicated group (OR 0.35; 95% CI, 0.32–0.38), a difference illustrated in Figure 2. Return visits to the ED occurred in 19.8% of intoxicated patients and 18.3% of non-intoxicated patients at 30 days (OR 1.10; 95% CI, 1.05–1.15), increasing to 45.8% and 42.1%, respectively, at six months (OR 1.16; 95% CI, 1.12–1.20).

**Figure 3:**
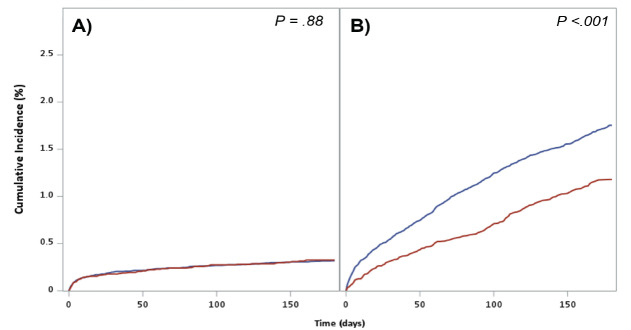
Cumulative incidence curves for six-month suicide-specific (A) and all-cause mortality (B) following emergency department (ED) presentation for suicidal behavior, stratified by alcohol intoxication status. Blue line indicates non-intoxicated patients while red line indicates intoxicated patients. Graphs are based on data from 58,051 patients across 16 EDs in Alberta, Canada (2011–2021).

Mortality outcomes at six months are shown in Table 2. A total of 0.3% of patients in both the intoxicated and non-intoxicated groups died by suicide (OR 1.03; 95% CI, 0.75–1.40), and the median time from the index ED visit to death by suicide was similar between groups (106 days [IQR 57–142] for intoxicated patients compared to 111 days [IQR 60–145] for non-intoxicated patients). All-cause mortality was 1.5% in the intoxicated group vs 2.1% in the non-intoxicated group (OR 0.72; 95% CI 0.63–0.83). Figure 3 presents the cumulative incidence curves for suicide-specific and all-cause mortality, with no statistically significant difference observed between the two groups in the death-by-suicide group but a significant early separation over the 180-day follow-up period for the all-cause mortality group. Supplementary Table 1 provides more extensive subgroup breakdowns for death by suicide and time to death while Supplementary Table 2 provides the subgroup unadjusted and adjusted HRs.

## DISCUSSION

### Key Findings

In this large, province-wide study of ED patients presenting with suicidal behaviors, acute alcohol intoxication was not associated with a significantly higher six-month risk of suicide-specific mortality compared to those who were not intoxicated. This finding suggests that intoxication may not elevate suicide-specific risk beyond the already high baseline in this population. Although this finding might initially appear to contrast with the extensive literature linking chronic AUD to elevated suicide risk,^3^,^5^–^7^ it offers a more nuanced view of acute intoxication in the ED setting. This should not be interpreted as diminishing the well-established association between chronic AUD and suicide risk in the community. Instead, this finding is a reflection that in the acute ED context, intoxication does not appear to add additional short-term risk beyond an already high baseline. In particular, these results are consistent with studies indicating that acute alcohol use, in isolation, may not necessarily translate into increased short-term mortality.^10^,^13^ Rather than negating the established risks posed by chronic AUD, these data supplement existing knowledge by highlighting that the interplay between acute intoxication, ongoing alcohol misuse, and suicide risk may be more complex than previously recognized.

One possible explanation is that individuals who arrive intoxicated often undergo prolonged observation (including involuntary holds) to ensure they are cooperative and cognitively intact for a thorough psychiatric assessment.^23^ In this study nearly one-fourth of intoxicated patients were placed under a hold, yet many of these patients were ultimately discharged rather than admitted. These findings coincide with previous studies examining outcomes of intoxicated patients placed under involuntary holds.^24^ It is plausible that intensive ED-based observation or acute interventions—such as brief crisis counseling or safety planning—could mitigate immediate suicide risk, particularly within the first few months post-ED visit.

### Emergency Department Psychiatry Consultations and the Role of Emergency Physicians

In this cohort, most patients presenting to the ED for suicidal ideation or suicide attempt were managed without an in-ED psychiatry consultation. The proportion of psychiatric consults, however, was higher for those presenting with acute alcohol intoxication (15.8%) compared to the non-intoxicated group (12.6%). This small but significant difference is hypothesized to reflect a growing recognition among Canadian emergency physicians of the mortality impact of AUD on suicidality and highlights the physician’s pivotal role in mental health management. Earlier research has raised concerns that acute alcohol intoxication might reduce the likelihood of an ED psychiatry consultation;^25^ however, these data suggest a shift toward more frequent referral when intoxication is documented.

### All-Cause Mortality and Potential Misclassification

When broadening the analysis to include all-cause mortality, we observed that patients who presented without acute alcohol intoxication were more likely to die in the subsequent six months. Although the overall six-month all-cause mortality for our cohort was 1.9%, this was only marginally higher than the background rate of 1.82% in the general ED population over the same period for ED patients in Alberta. This finding suggests that, even among a high-risk group such as those presenting with suicidality or self-harm, the absolute mortality risk remains close to that of typical ED patients. While this finding may reflect unmeasured confounders, such as chronic medical comorbidities or different patterns of substance misuse, it also raises questions about potential misclassification in cause-of-death reporting.^26^ It is possible that some suicides in the non-intoxicated group were recorded under alternative categories, or that these patients succumbed to other conditions triggered or exacerbated by their suicidal crisis. Similar trends have been reported in previous population-based studies,^27^ suggesting that temporal proximity to an ED visit for suicidality may be indicative of elevated risk for non-suicide external causes of death. Further investigation into the accuracy and methodology of provincial and federal suicide reporting systems could help clarify the true burden of suicide-related mortality in this population.

### Extended Emergency Department Length of Stay and Recurrent Visits

Our results also confirm that acutely intoxicated patients with suicidality consume substantial ED resources. They had significantly longer LOS and were more likely to revisit the ED within 30 days and six months. Previous work has documented similar resource use patterns among patients requiring mental health evaluation while intoxicated,^25^,^28^ as well as those with alcohol involvement in trauma.^29^ These findings reinforce the importance of targeted ED interventions, such as motivational counseling or linkage to addiction services, to reduce repeat visits and address longer term risks.

While our main analysis did not isolate suicidal ideation-only visits, we found that 1.2% of patients in the cohort had an ED visit for suicidal ideation within six months prior to their index presentation for self-harm or suicide attempt. This pattern was more common among patients who were intoxicated at the time of their index visit (1.5% vs 1.0%). Although a small proportion overall, this subgroup may represent individuals with recurring risk and comorbid substance use. The association between prior suicidal ideation presentations and intoxication at the time of a subsequent attempt highlights the potential importance of interventions for substance use disorder on an outpatient basis.

### Acute vs Chronic Alcohol Use

It remains important to distinguish between acute intoxication and chronic AUD. Long-standing AUD is well known to increase lifetime suicide risk,^5^–^7^ whereas this study specifically focused on ED presentations marked by acute intoxication. The absence of a clear mortality difference for suicide-specific death may indicate that the acute episode itself is not strongly associated with suicide within six months, or that ED-based safety measures could mitigate this risk. Nevertheless, repeated intoxication episodes and ongoing suicidal ideation could still amplify longer term risk beyond the scope of this study’s follow-up period. Our findings, therefore, complement, rather than challenge, the extensive literature linking AUD to suicide risk.

### Strengths

This is one of the largest population-based studies to date examining the intersection of acute alcohol intoxication and suicidality in patients presenting to the ED. By focusing on mortality outcomes and ED management patterns, this study addresses a significant gap in understanding the association between acute intoxication, post-discharge mortality rates, and ED resource utilization.

### Implications and Future Directions

For emergency clinicians, these findings suggest that patients arriving with acute intoxication and suicidal behavior do not, in isolation, face a higher short-term risk of death by suicide than their non-intoxicated counterparts. However, both groups remain at elevated risk compared to the general population. System-level strategies remain essential, however, to address potential chronic AUD, comorbid psychiatric conditions, and the need for robust post-discharge planning. Future research should investigate whether substance use-related ED interventions such as brief motivational interviewing or urgent referrals to addiction services can reduce suicide or all-cause mortality.

### Involuntary Holds in Alberta

In the Alberta healthcare system, the process for involuntary psychiatric holds may differ from other locales. Patients typically arrive either by ambulance or by self-presentation. Those arriving by ambulance may be doing so under a Form 10, an involuntary apprehension issued by police services that compels transfer to the ED for assessment. These patients cannot leave until they are evaluated by a physician, who must then determine whether the criteria for issuing a Form 1 (an involuntary hold permitting detention for up to 24 hours for psychiatric assessment) are met. If not, the Form 10 is rescinded and the patient regains the right to leave voluntarily.

Any patient who self-presents to the ED, including those with suicidal ideation and/or alcohol intoxication, are first triaged by nursing staff and placed in the general waiting area until an assessment room or stretcher becomes available. There is no mechanism to automatically detain these patients prior to a physician’s assessment, and until that assessment occurs they are legally free to leave the ED. Additionally, those arriving by ambulance also may be doing so voluntarily and, thus, there are no legal grounds to detain them in the ED until a physician assesses them to determine whether they warrant a Form 1 being issued.

The leaving without being seen (LWBS) and leaving against medical advice (LAMA) outcomes in our dataset reflect these operational nuances. Leaving without being seen refers to patients who arrive to the ED but depart before any physician contact, while LAMA applies to those who have been assessed by a physician but choose to leave before care is completed. In both instances, physicians must balance patient safety with the legal threshold for involuntary certification and revoking a person’s autonomy. For example, a patient presenting with suicidal ideation may experience a reduction in acute risk after observation or crisis counseling, making continued detention unwarranted. While the physician may disagree with them leaving before receiving additional consultation, they cannot involuntarily hold the patient in the ED for this treatment if they do not meet the criteria delineated in a Form 1.

Understanding these distinctions is essential when interpreting the rates of LWBS and LAMA observed in our cohort. These events do not necessarily indicate deficiencies in care or oversight but rather reflect the legal and ethical framework governing psychiatric certification in Alberta, which emphasizes patient rights and proportionality in the use of involuntary holds.

## LIMITATIONS

This study has several limitations. First, the use of *ICD-10-CA* codes to identify suicide attempts, self-harm, and acute alcohol intoxication may result in underestimation or misclassification, given that these codes often have modest sensitivity even if specificity is relatively high. Second, key variables such as psychiatric diagnoses, prior suicide attempts, and other substance use disorders were not consistently captured in the administrative data, potentially introducing unmeasured confounding. Third, because the study was conducted in a single Canadian province, the findings may not be generalizable to jurisdictions with different legal frameworks for involuntary holds, varying firearm availability, or distinct cultural attitudes toward alcohol and suicide.

Fourth, while alcohol is an immediately detectable substance of misuse tested commonly in the ED environment, other drug screening is rare in the ED setting given the time delay between collection and reporting and the cost of testing the multiple drugs that affect behavior. Finally, the study’s observational design precludes causal inferences, as the observed associations may reflect residual confounding rather than a direct effect of acute intoxication. Nevertheless, by leveraging a comprehensive provincial database, this study was able to capture diverse ED settings and reliably ascertain mortality outcomes within the critical six-month period following an ED visit for suicidal behavior.

## CONCLUSION

In a large cohort of ED patients presenting with suicidal behavior, acute alcohol intoxication at the time of presentation was not associated with a higher risk of suicide-specific death over six months. These findings should be interpreted within the ED context and do not diminish the established role of chronic AUD as a major risk factor for suicide in community settings. Clinicians caring for intoxicated, suicidal patients should continue to monitor and manage these patients carefully, given their high rates of involuntary holds, extended ED stays, and frequent recidivism. Future research should explore the influence of underlying psychiatric comorbidities and other substances on suicide risk, as well as interventions to link these high-risk individuals to outpatient services for both mental health and potential alcohol use disorder treatment.

## Supplementary Information





## Figures and Tables

**Table 1:** Characteristics of patients presenting to the emergency department with suicidal behavior, stratified by alcohol intoxication status, in a retrospective cohort study of 58,051 patients across 16 EDs in Alberta, Canada (2011–2021).

	Total N = 58,051	Patients intoxicated with alcohol n = 17,488	Patients not intoxicated with alcohol n = 40,563	Unadjusted OR (95% CI) or median differences (95% CI)
Age (years), median (IQR)*	34 (24, 48)	33 (24, 47)	34 (24, 49)	1.00 (0.73 – 1.27)
Male sex (n [%])	31,056 (53.5)	10,058 (57.5)	20,998 (51.8)	1.26 (1.22 – 1.31)
CTAS score (n [%])				
1 – resuscitation	4,049 (7.0)	1,658 (9.5)	2,391 (5.9)	1.80 (1.68 – 1.94)
2 – emergent	31,298 (53.9)	9,962 (57.0)	21,336 (52.6)	1.22 (1.17 – 1.27)
3 – urgent	16,821 (29.0)	4,671 (26.7)	12,150 (30.0)	Ref
4 – less urgent	5,354 (9.2)	1,131 (6.5)	4,223 (10.4)	0.70 (0.65 – 0.75)
5 – non-urgent	520 (0.9)	61 (0.3)	459 (1.1)	0.35 (0.26 – 0.45)
Missing	9 (0.0)	5 (0.0)	4 (0.0)	-
Arrived by ambulance (n [%])				
Ambulance	32,193 (55.5)	12,136 (69.4)	20,057 (49.4)	2.32 (2.23 – 2.41)
No ambulance	25,858 (44.5)	5,352 (30.6)	20,506 (50.6)	0.43 (0.42 – 0.45)

Note:

* 3 missing data.

*CTAS*, Canadian Triage and Acuity Scale; *OR*, odds ratio.

**Table 2:** Emergency department (ED) factors and post-discharge outcomes among patients presenting with suicidal behavior, stratified by alcohol intoxication status, in a retrospective cohort study of 58,051 patients across 16 EDs in Alberta, Canada (2011–2021).

	Total N = 58,051	Patients intoxicated with alcohol n = 17,488	Patients not intoxicated with alcohol n = 40,563	Unadjusted OR (95% CI) or median differences (95% CI)
Health service outcomes (n [%])				
Form 1 billing by cliniciansα	11,005 (19.0)	4,613 (26.4)	6,392 (15.8)	1.92 (1.83 – 2.00)
Any consultations	15,770 (27.2)	4,688 (26.8)	11,082 (27.3)	0.97 (0.94 – 1.01)
Psychiatric consultation	7,858 (13.5)	2,764 (15.8)	5,094 (12.6)	1.31 (1.24 – 1.37)
Disposition (n [%])				
Discharged	44,825 (77.2)	14,525 (83.1)	30,300 (74.7)	Ref
Admitted	8,121 (14.0)	1,890 (10.8)	6,231 (15.4)	0.63 (0.60 – 0.67)
Transferred	1,862 (3.2)	463 (2.6)	1,399 (3.4)	0.69 (0.62 – 0.77)
LWBS	1,975 (3.4)	259 (1.5)	1,716 (4.2)	0.32 (0.28 – 0.36)
LAMA	1,268 (2.2)	351 (2.0)	917 (2.3)	0.80 (0.71 – 0.90)
ED length of stay in minutes, median (IQR)	317 (181, 544)	411 (255, 640)	277 (158, 486)	−134.0 (−140.3 to −127.7)
Return ED visits within 30 days (n [%])	10,902 (18.8)	3,468 (19.8)	7,434 (18.3)	1.10 (1.05 – 1.15)
Number of ED visits, median (IQR)	1 (1, 2)	1 (1, 2)	1 (1, 2)	-
Return ED visits within 30 days due to suicide attempt	1,841 (3.2)	605 (3.5)	1,236 (3.0)	1.14 (1.03 – 1.26)
Number of ED visits, median (IQR)	1 (1, 1)	1 (1, 1)	1 (1, 1)	-
Return ED visits within 6 months (n [%])	25,106 (43.2)	8,013 (45.8)	17,093 (42.1)	1.16 (1.12 – 1.20)
Number of ED visits, median (IQR)	2 (1, 3)	2 (1, 3)	2 (1, 3)	-
Return ED visits within 6 months due to suicide attempt	5,005 (8.6)	1,800 (10.3)	3,205 (7.9)	1.34 (1.26 – 1.42)
Number of ED visits, median (IQR)	1 (1, 1)	1 (1, 1)	1 (1, 1)	-
Death within 6 months (all-cause) (n [%])	1,102 (1.9)	263 (1.5)	839 (2.1)	0.72 (0.63 – 0.83)
Death within 6 months due to suicide (n [%])	186 (0.3)	57 (0.3)	129 (0.3)	1.03 (0.75 – 1.40)
Time from first presentation to death by suicide, days, median (IQR)	17.5 (3, 68)	21 (3, 82)	16 (3, 66)	−5.0 (−25.8 to 15.8)

α Specialties for physicians with Form 1 claim overlapping with ED visit include full-time emergency physician, emergency medicine – specialty, psychiatry – specialty, general practice, and generalists mental health physicians (ED setting only).

“Form 1” refers to an involuntary mental health hold under the provincial Mental Health Act.

*ED*, emergency department; *LAMA*, leave against medical advice; *LWBS*, leave without being seen;, odds ratio.
