# Supplementary material for: Impact of Alcohol Intoxication on Mortality and Emergency Department Resource Use in Suicidal Patients
Source: West J Emerg Med. 2026 Jan 3;27(1):104–13. doi: 10.5811/westjem.48788 (PMC12815556; doi:10.5811/westjem.48788)
Supplement: Supplementary file 1 [file wjem-27-104-s001.docx]

**Supplementary Table** - Psychiatric consultation, admission rates, suicide-specific mortality, and time to death (in days from index visit) stratified by alcohol intoxication and legal hold status, in a retrospective cohort study of 58,051 patients presenting with suicidal behavior across 16 EDs in Alberta, Canada (2011–2021).

|  | Alcohol Intoxicated Group | | | | | | | |
| --- | --- | --- | --- | --- | --- | --- | --- | --- |
|  | Involuntary Hold  n=4,613 (26.4%) | | | | No Involuntary Hold  n=12,875 (73.6%) | | | |
|  | Psychiatry Consult | No Psychiatry Consult | Admission | No Admission | Psychiatry Consult | No Psychiatry Consult | Admission | No Admission |
| n, (%) | 1975 (42.8) | 2638 (57.2) | 856 (18.6) | 3757 (81.4) | 789 (6.1) | 12086 (93.9) | 1034 (8.0) | 11841 (92.0) |
| Death by Suicide, n (%) | 7 (0.4) | 11 (0.4) | 7 (0.8) | 11 (0.3) | 5 (0.6) | 34 (0.3) | 23 (2.2) | 16 (0.1) |
| Time to death, median (IQR) | 142 (85, 151) | 47 (9, 68) | 53 (2, 156) | 82 (26, 142) | 34 (14, 59) | 6 (3, 41) | 4 (2, 7) | 44 (17, 78) |

|  | Non-Intoxicated Group | | | | | | | | | |
| --- | --- | --- | --- | --- | --- | --- | --- | --- | --- | --- |
|  | Involuntary Hold  n=6,392 (15.8%) | | | | | No Involuntary Hold  N=34,171 (84.2%) | | | | |
|  | Psychiatry Consult | No Psychiatry Consult | Admission | No Admission | Psychiatry Consult | | No Psychiatry Consult | Admission | No Admission |  |
| n, (%) | 3300 (51.6) | 3092 (48.4) | 2536 (39.7) | 3856 (60.3) | 1794 (5.3) | | 32377 (94.8) | 3695 (10.8) | 30476 (89.2) |  |
| Death by Suicide, n (%) | 13 (0.4) | 16 (0.5) | 20 (0.8) | 9 (0.2) | 9 (0.5) | | 91 (0.3) | 63 (1.7) | 37 (0.1) |  |
| Time to death, median (IQR) | 66 (21, 88) | 56 (22, 102) | 62 (19.5, 102) | 53 (27, 80) | 53 (28, 81) | | 8 (2, 32) | 4 (2, 22) | 31 (9, 84) |  |
