# Supplementary material for: Impact of Alcohol Intoxication on Mortality and Emergency Department Resource Use in Suicidal Patients
Source: West J Emerg Med. 2026 Jan 3;27(1):104–13. doi: 10.5811/westjem.48788 (PMC12815556; doi:10.5811/westjem.48788)

Supplementary Table 2 – Hazard ratio (with 95% confidence intervals) from subdistribution hazard model for death. aHR represents the adjusted hazard ratio for the subdistribution hazard model, where death from suicide is the outcome of interest and all-cause death is a competing risk. Data from a retrospective cohort study of 58,051 patients presenting with suicidal behavior across 16 EDs in Alberta, Canada (2011–2021).


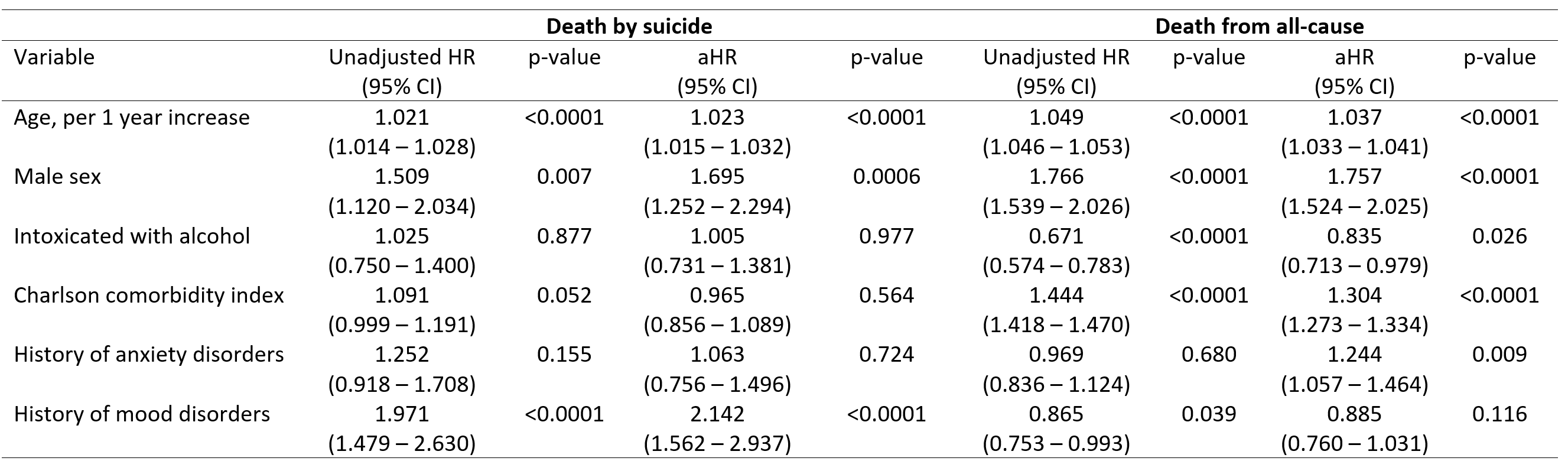

Supplement: Supplementary file 2 [file wjem-27-104-s002.docx]
